# Comparison of 1064-nm Nd:YAG picosecond laser using fractional micro-lens array vs. ablative fractional 2940-nm Er:YAG laser for the treatment of atrophic acne scar in Asians: a 20-week prospective, randomized, split-face, controlled pilot study

**DOI:** 10.3389/fmed.2023.1248831

**Published:** 2023-11-16

**Authors:** Ru Dai, Yiyu Cao, Yiping Su, Suiqing Cai

**Affiliations:** ^1^Department of Dermatology, The Second Affiliated Hospital, Zhejiang University School of Medicine, Hangzhou, Zhejiang, China; ^2^Research Department of Industrial Development, Zhejiang Development & Planning Institute, Hangzhou, China; ^3^Department of Dermatology, The First People Hospital of Hangzhou Linan District, Hangzhou Medical College, Hangzhou, Zhejiang, China

**Keywords:** acne scar, picosecond laser, fractional laser ablation, laser treatment, efficacy and safety

## Abstract

**Background:**

The 1064-nm Nd:YAG picosecond lasers using fractional micro-lens array (P-MLA) was a promising therapy for skin resurfacing. However, no studies have compared P-MLA with ablative fractional 2940-nm Er:YAG lasers (AF-Er) in the treatment of atrophic acne scars.

**Objectives:**

To evaluate the efficacy and safety of P-MLA and AF-Er for the treatment of atrophic acne scars.

**Methods:**

We performed a prospective, randomized, split-face, controlled pilot study. Thirty-one Asian patients with mild to moderate atrophic acne scars underwent four consecutive sessions of randomized split-face treatment with P-MLA and AF-Fr at 4-week intervals. The efficacy of the two devices were evaluated by Echelle d’Evaluation Clinique des Cicatrices d’acne (ECCA) grading scale, Investigator’s Global Assessment (IGA) score and patient’s satisfaction. VISIA analysis was also performed to evaluate the pore and skin texture. Adverse events were recorded at each follow-up.

**Results:**

The P-MLA afforded comparable clinical responses in scar appearance as AF-Er based on the investigator’s assessments (ECCA percent reduction: 39.11% vs. 43.73%; IGA score: 2.97 ± 0.65 vs. 3.16 ± 0.68; *P* > 0.05 for both). However, the result of patient satisfaction indicated the AF-Er-treated side achieved a slightly greater improvement in scar appearance (3.97 ± 0.78 vs. 3.55 ± 0.71; *P* < 0.05). Overall, the two devices did not differ largely in terms of efficacy. VISIA analysis revealed similar changing patterns of the pore and skin texture between two devices. For safety profiles, no serious side effects were reported on both sides. The P-MLA showed lower pain level, shortened duration of crust shed and edema, and less occurrence of PIH (*P* < 0.05 for all).

**Conclusion:**

Compared with AF-Er, P-MLA afforded comparable effect and more safety profiles in treating atrophic acne scars in Asian patients.

**Clinical trial registration:**

ClinicalTrials.gov, identifier NCT 05686603.

## 1. Introduction

Acne is a common skin disorder affecting 9.38% of the global population (1), especially among adolescents and young adults. Approximately 85% of teenagers experience acne at some points, and most of them can persist into adulthood (2). The prevalence of acne in adult women and men is about 18 and 8% respectively (3). Although acne is not life-threatening, the abnormal healing procedures of pilosebaceous ducts may result in scarring. One publication showed that 43% of cases with facial acne could develop scars (4), and the acne-associated scarring often has a negative effect on patients’ psychosocial and physical wellbeing. There are three general types of acne scars namely atrophic scars, hypertrophic scars and keloid scars. Among them, the majority of scars are atrophic which are seen in over 80% of patients (5). These scars present clinically as depressions in the skin surface due to destructive inflammatory process and decreased collagen deposition in the dermis (6).

A wide range of interventions have been proposed to treat atrophic acne scars, including laser, chemical peels, dermabrasion, injectable fillers and surgical methods (7). Among them, fractional ablative lasers such as 2940-nm erbium yttrium aluminum garnet (Er:YAG) laser and carbon dioxide (CO_2_) laser are the most commonly used treatments for atrophic acne scars (8). Ablative lasers can remove the damage tissue of the scars and allow collagen remodeling and re-epithelialization (9). However, the laser treatments may result in adverse effects including erythema, changes in pigmentation, scarring, infections and prolonged recovery period, especially in dark-skinned population, which limits their use in Asian patients who are usually Fitzpatrick skin types III to IV and desire minimal risks (5).

Picosecond laser is a novel technology characterized by ultra-short, picosecond pulse duration which can be effective for many skin conditions, such as pigmentation, photoaging and wrinkles reduction (10–13). When combined with micro-lens array (MLA) optics, high-intensity, micro-injury zones can be generated in the epidermis and dermis, causing optical breakdown of surrounding tissue and stimulating of dermal remodeling with mild side-effects (14). Only transient erythema, edema, hyperpigmentation, and tolerable pain were noted, which spontaneously resolve within a few days (15–17). No permanent side effects were reported. Previous studies had showed the picosecond lasers with MLA afforded better or similar clinical outcomes as well as fewer side-effects in treating acne scar than non-ablative lasers (18–20). However, the clinical improvements of non-ablative lasers for acne scar were not as impressive as the results from those using ablative laser resurfacing (6, 9). Nevertheless, there are insufficient prospective comparative studies evaluating the picosecond laser with MLA optics vs. current fractional ablative techniques for the treatment of atrophic acne scars.

In this study, we reported a prospective, randomized, split-face, controlled pilot study that comparing the efficacy and safety of a fractional 1064-nm neodymium-doped yttrium aluminum garnet (Nd:YAG) picosecond laser with MLA handpiece (P-MLA) and ablative fractional 2940-nm Er:YAG laser (AF-Er) for the treatment of atrophic acne scars in Asians.

## 2. Materials and methods

### 2.1. Study design

This prospective, randomized, split-face, controlled pilot study was approved by the Human Ethics Committee of Zhejiang University School of Medicine Second Affiliated Hospital (2022-0419) and performed in accordance with the Declaration of Helsinki. Written informed consent was obtained from all subjects before enrollment. The study was conducted at the outpatient dermatology department of Zhejiang University School of Medicine Second Affiliated Hospital from April 2022 to October 2022.

### 2.2. Patient selection

A total of thirty-three subjects (16 males and 17 females) aged above 18 years of Fitzpatrick skin types II to type V, with mild to moderate atrophic acne scars were recruited for this study. Because of the lack of previous data, we performed an exploratory study with a small sample. The sample size was determined based on previous feasibility studies rather than a power analysis. Inclusion criteria for this study were as follows: (1) age ≥18 years; (2) presence with similar atrophic acne scars on both sides of the face; and (3) signed informed consent and cooperated with the follow up and complied the study protocol. Subjects were excluded if they had active acne under treatments or had a previous history of keloid or hypertrophic scar formation, undergone any acne scar treatments or anesthetic treatments for the face in the past 6 months before the first treatment, were pregnant or lactating females, were sensitive to lights, were allergic to lidocaine, had other preexisting skin conditions or uncontrolled systemic diseases. However, if patients had active acne while were unwilling to take medical treatments, they were included. In addition, patients were prohibited to perform other cosmetology and aesthetic medicine treatments during this study.

### 2.3. Treatment

**FIGURE 1:**
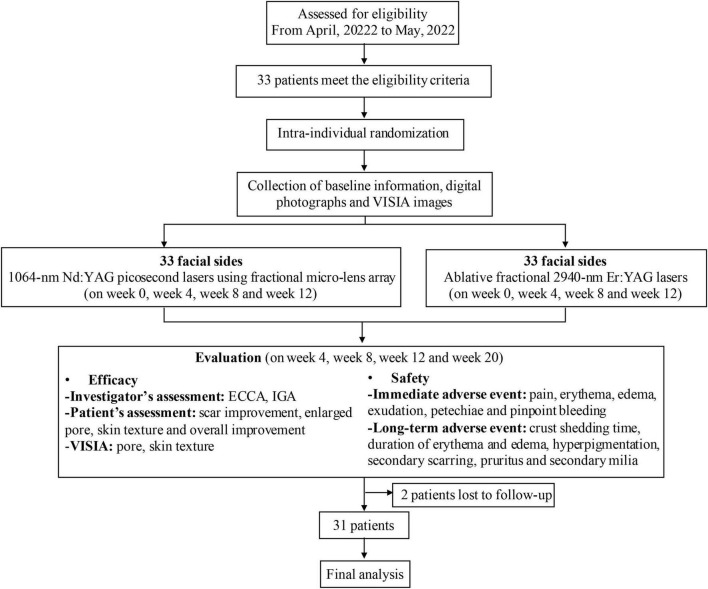
Flow diagram of the study. ECCA, Echelle d’Evaluation Clinique des Cicatrices d’acne; IGA, Investigator’s Global Assessment.

Enrolled participant was randomized to receive split-face treatment with fractional 1064-nm Nd:YAG picosecond lasers (Picocare™, Wontech, Republic of Korea) on one side and ablative fractional 2940-nm Er:YAG laser (Dermablate MCL31, Asclepion Laser Technologies, Germany) on the other side. The block randomization was used to assign the treatment modality. A digital photograph was taken with the same lighting and positioning consistency in each subject (Figure 1).

Enrolled subjects were informed not to wear makeup before each treatment. Topical anesthetic cream was applied 30 min before laser treatment. Patients cleansed their face with a mild cleanser. Then, one-half of the face was treated with P-MLA and the other half used AF-Er according to the randomly assigned allocation. The following parameters were used: the P-MLA with spot size of 7–10 mm, fluence of 0.8–1.4 J/cm^2^, pulse duration of 450-picosecond, and frequency of 10 Hz was applied for three to six passes, while the AF-Er with a model of N25% and energy density of 20-30 J/cm^2^ was applied for one to two passes. The N25% model covers 25% of treated area. Lasers were done over all scar area with up to 20% overlapping between the adjacent pulses. As for P-MLA side, immediate erythema and mild petechiae were regarded as the desired clinical endpoints. As for AF-Er side, the clinical endpoints were mild erythema and oozing of bloody serous exudates. After treatment, ice pack was applied for 20 min and antibiotic ointment was then prescribed on the treated areas. Each patient was instructed to avoid sun exposure for at least 2 weeks and to use a broad-spectrum sunscreen daily. Each patient received four consecutive laser sessions at an interval of 4 weeks, and was followed up 8 weeks after final treatment.

### 2.4. Assessment

Thorough history taking and physical examination were performed in all subjects. Efficacy and safety of the treatments were evaluated at each visit, and VISIA images (Visia CR^®^; Canfield Scientific, Parsippany, NJ, USA) of front, left and right face were also obtained at both baseline and last visit for final analyses.

#### 2.4.1. Efficacy

Efficacy of scar improvement was evaluated by investigators and patients. A blinded investigator assessed the clinical efficacy by the Echelle d’Evaluation Clinique des Cicatrices d’acne (ECCA) grading scale and Investigator’s Global Assessment (IGA) scores. ECCA score is calculated on the sum of the number and type of scar (V-type, U-type, and M-type) (21). IGA was evaluated using a 5-point scale as follows: 0 = no improvement; 1 = 1–25% improvement; 2 = 26–50% improvement; 3 = 51–75% improvement; 4 = 76–100% improvement. Patients rated their degree of satisfaction about scar improvement, pore, skin texture and overall improvement using a Likert satisfaction scale (1 = very dissatisfied, 2 = dissatisfied, 3 = slightly satisfied, 4 = satisfied, 5 = very satisfied). The primary endpoints were the change of ECCA score, IGA score and degree of patient’s satisfaction at the final visit compared the baseline score. We also used VISIA system to evaluate the pore and skin texture objectively.

#### 2.4.2. Safety

Patients were evaluated at each session immediately for adverse effects including pain, erythema, edema, exudation, pinpoint bleeding, and petechiae. The pain was evaluated using a visual analog scale (VAS) ranging from 0 (no pain) to 10 (unbearable pain). Other immediate adverse effects were recorded with a 0-to-3 severity scale (0 = none; 1 = mild; 2 = moderate; 3 = severe). At next follow-up, patients were also asked to record and document their recovery times and possible long-term adverse effects, including crust shedding time, duration of erythema and edema, post-inflammatory hyperpigmentation (PIH), scarring formation, pruritus and acneiform eruption.

### 2.5. Statistical analysis

All statistical analyses were performed on SPSS for Mac software (version 26.0) and GraphPad Prism 9 (version 9.2.0). Descriptive data were presented as mean values with standard deviation (SD). Paired samples *t*-test was used to compare before and after treatment as well as the clinical outcomes between P-MLA and AF-Er. When paired *t*-test was not satisfied, Wilcoxon matched-pairs singed rank test was used to determine subjects’ assessment of the effectiveness and satisfaction score for different treatments. A *P*-value < 0.05 was considered statistically significant and all probability values were two-tailed.

## 3. Results

### 3.1. Patient characteristics

Thirty-one subjects completed the study, and two patients dropped out at 4 weeks after first treatment because of job transfer and COVID-19 respectively. None of the remaining thirty-one patients withdrew due to treatment-related adverse events. There was no significant difference in mean ECCA score at baseline between two facial sides.

Baseline patient demographic characteristics are shown in Table 1. The mean age of participants was 26.48 ± 3.06 years. Seventeen participants (54.84%) were female and fourteen (45.16%) were male. Among them, most patients were categorized as having Fitzpatrick skin type IV (15 patients, 48.39%) or type III (13 patients, 41.94%). The rest consisted of type II in 1 patient (3.23%) and type V in 2 patients (6.45%). The average duration of acne scarring was 9.71 ± 2.61 years with age ranging from 20 to 32 years old. For subtype of atrophic scars, most patients (23 subjects, 74.19%) had a combination of all three types of scars. Only one patient had U type scars only. The rest (7 subjects, 22.58%) were a mixture of V-shaped and U-shaped scars.

**TABLE 1:** Baseline characteristics.

Characteristics	(*N* = 31)
Age (year), mean ± SD	26.48 ± 3.06
BMI (kg/m^2^), mean ± SD	21.96 ± 3.14
**Gender, *n* (%)**	
Female	17 (54.84)
Male	14 (45.16)
**Fitzpatrick skin type, *n* (%)**	
Type II	1 (3.23)
Type III	13 (41.94)
Type IV	15 (48.39)
Type V	2 (6.45)
Duration of scars (year), mean ± SD	9.71 ± 2.61
**Baseline scar type, *n* (%)**	
V type	0 (0)
U type	1 (3.23)
W type	0 (0)
V + U type	7 (22.58)
V + W type	0 (0)
U + W type	0 (6.45)
V + U + W type	23 (74.19)

SD, standard deviation.

### 3.2. Assessment of efficacy

**FIGURE 2:**
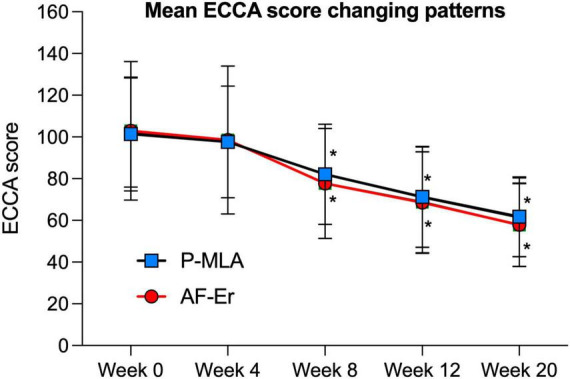
Mean ECCA scores of each side evaluated from baseline to the final observation. Error bars represent SDs. ECCA, Echelle d’Evaluation Clinique des Cicatrices d’acne; P-MLA, picosecond lasers with MLA handpiece; AF-Er, ablative fractional 2940-nm Er:YAG laser. *P < 0.05 compared with the baseline.

**FIGURE 3:**
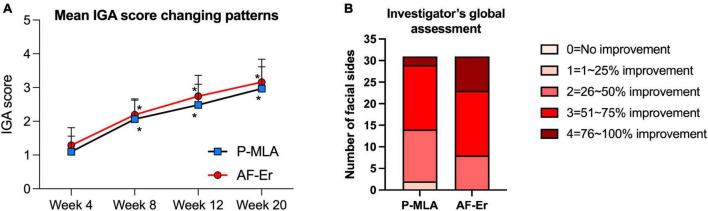
Evaluation of scar improvement based on IGA score. **(A)** Mean IGA scores of two facial sides from first treatment to the final observation. *P < 0.05 compared with the first treatment. **(B)** Evaluation of scar improvement at final follow-up. IGA, investigator’s global assessment.

**FIGURE 4:**
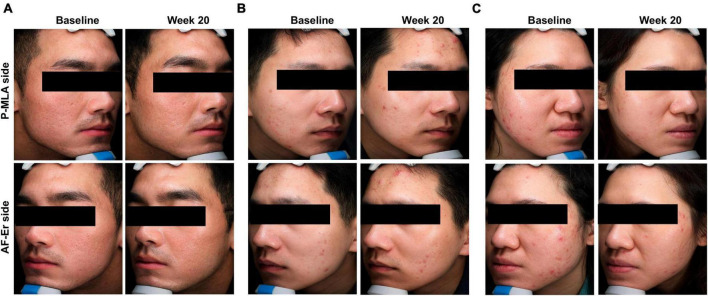
Clinical photographs revealed the improvement of acne scars by both treatments. **(A)** A 23-year-old man with Fitzpatrick skin Type IV. **(B)** A 27-year-old man with Fitzpatrick skin Type IV. **(C)** A 22-year-old women with Fitzpatrick skin Type IV. P-MLA, picosecond lasers with MLA handpiece; AF-Er, ablative fractional 2940-nm Er:YAG laser.

The ECCA scores evaluated from baseline to the final observation were shown in Figure 2. Both lasers could improve acne scars significantly after four sessions of treatment, while the effects of two devices were not statistically significant in ECCA score at each visit. For the P-MLA side, the ECCA score decreased from 101.45 ± 27.33 at baseline to 61.77 ± 19.03 at the final visit (*P* < 0.05) with an average reduction of 39.11%. As for the AF-Er sides, the ECCA score decreased from 102.90 ± 33.23 to 57.90 ± 20.03 (*P* < 0.05) with an average reduction of 43.73%. Moreover, a significant reduction of ECCA score from the baseline was observed after two treatments by both devices (*P* < 0.05). A further subgroup analysis of scar subtypes was performed to evaluate potential differences (Table 2). Similarly, both P-MLA and AF-Er showed significant improvement in V-type, U-type and M-type scars (*P* < 0.05 for all). However, the improvements of different scars by two devices were out of significance. The IGA score of scar improvement demonstrated a similar pattern with ECCA and the detailed information is presented in Figure 3. Both P-MLA and AF-Er could significantly improve acne scars after four sessions of treatment. Specifically, the IGA score ± SD increased from 1.10 ± 0.47 at the first follow-up to 2.97 ± 0.65 at the final visit on the P-MLA side, and from 1.29 ± 0.52 to 3.16 ± 0.68 on the AF-Er side. The average IGA score of AF-Er-treated side were higher than those of P-MLA-treated side at each visit, while no significant difference was observed (all *P* > 0.05). At the final visit, seventeen out of 31 (54.84%) facial sides achieved grade 3 or more improvements after P-MLA treatment, and 23 out of 31 (74.19%) facial sides did after AF-Er treatment (*P* = 0.09). The representative cases were shown in Figure 4.

**TABLE 2:** Echelle d’Evaluation Clinique des Cicatrices d’acne (ECCA) score for V, U, and M subtypes of scar.

ECCA score	P-MLA side	AF-Er side	*P*-value
**V-type scar**			*P* = 0.66
Baseline	38.23 ± 10.84	37.74 ± 10.15	
Week 20	26.13 ± 8.63	24.67 ± 8.13	
	*P* < 0.05*	*P* < 0.05*	
**U-type scar**			*P* = 0.10
Baseline	38.67 ± 11.67	40.67 ± 14.37	
Week 20	30.67 ± 11.43	27.33 ± 11.12	
	*P* < 0.05*	*P* < 0.05*	
**M-type scar**			*P* = 0.73
Baseline	34.78 ± 16.41	34.57 ± 16.58	
Week 20	28.57 ± 9.45	28.13 ± 8.84	
	*P* < 0.05*	*P* < 0.05*	

ECCA, Echelle d’Evaluation Clinique des Cicatrices d’acne; P-MLA, picosecond lasers with MLA handpiece; AF-Er, ablative fractional 2940-nm Er:YAG laser. *P < 0.05 compared with the baseline.

**FIGURE 5:**
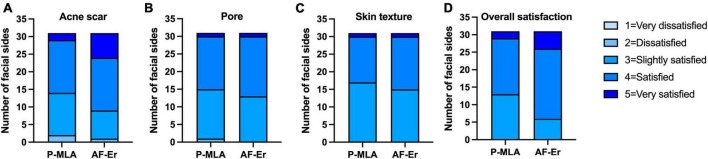
Patients’ subjective assessment of scar improvement. Patients’ subjective assessment at the final observation for the improvement of acne scar **(A)**, pore **(B)**, skin texture **(C)**, and overall satisfaction **(D)** using Likert satisfaction scale (1 = very dissatisfied, 2 = dissatisfied, 3 = slightly satisfied, 4 = satisfied, 5 = very satisfied). P-MLA, picosecond lasers with MLA handpiece; AF-Er, ablative fractional 2940-nm Er:YAG laser.

Figure 5 shows the efficacy assessed by patients. As for scar improvement (Figure 5A), patients were more satisfied with AF-Er. The proportion of patients whose subjective self-ratings reported satisfied or very satisfied was 17 out of 31 (54.83%) on the P-MLA side and 22 out of 31 (70.97%) on the AF-Er side, while the difference was out of significance (*P* = 0.15). However, the mean satisfaction score was significantly higher on the AF-Er side than the P-MLA side (3.97 ± 0.78 vs. 3.55 ± 0.71 on a five-point Likert satisfaction scale; *P* < 0.05). Regarding the enlarged pores (Figure 5B), the degrees of subjective satisfaction between the two treatments presented comparable tendencies (P-MLA vs. AF-Er: 51.61 vs. 58.06%; *P* = 0.40). As for skin texture (Figure 5C), the proportion of patients who self-rated satisfied or very satisfied was 14 out of 31 (45.16%) on the P-MLA side and 16 out of 31 (51.61%) on the AF-Er side (*P* = 0.40). Furthermore, subjective assessment of the overall improvement as satisfied to very satisfied was 80.65% with AF-Er treatment, which was significantly superior than that on the P-MLA side (58.96%; *P* < 0.05) (Figure 5D).

**FIGURE 6:**
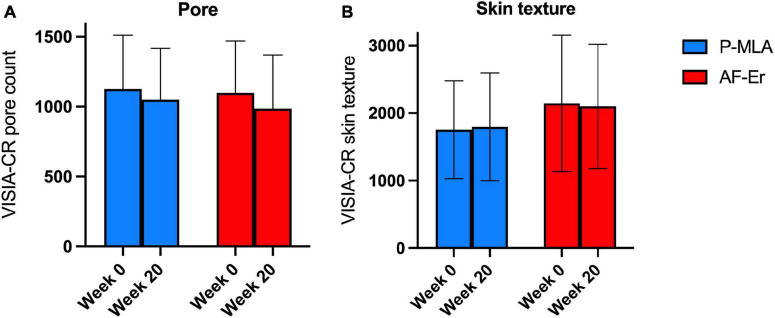
Results from the VISIA system. **(A)** Pore count of two facial sides from baseline through 20-week follow-up visit. **(B)** Skin texture value of two facial sides from baseline through 20-week follow-up visit. P-MLA, picosecond lasers with MLA handpiece; AF-Er, ablative fractional 2940-nm Er:YAG laser.

Image analysis based on the VISIA system at baseline and the final visit showed reduction of pore counts by both devices (Figure 6A), while the differences were out of significance (both *P* > 0.05). The values of measuring the skin texture showed similar counts before and after the treatments (Figure 6B), and no obvious changing pattern was observed neither on the P-MLA side nor on the AF-Er side.

### 3.3. Assessment of safety

No serious side effects were reported through the duration of the study (Table 3). Considering the immediate adverse effects, the mean VAS pain score on the P-MLA side were 2.14 ± 1.00, which was significantly lower than that of the AF-Er side (3.53 ± 1.30; *P* < 0.05). Erythema was transient and seen on both treated sides, while the mean scale for post-treatment erythema were more severe on the P-MLA side than on the AF-Er side (1.12 ± 0.70 vs. 0.94 ± 0.60; *P* < 0.05). Edema and pinpoint bleeding were experienced by some patients. The incidence of edema was 6.45% on the P-MLA side and 12.90% on the AF-Er side (*P* = 0.34), and pinpoint bleeding was observed in 29.03 and 38.71% facial sides treated with P-MLA and AF-Er (*P* = 0.30), respectively. Exudation was more common on the AF-Er side and was observed on 96.77% patients, the occurrence of which was significantly higher than that on the P-MLA side (6.45%; *P* < 0.05). As for petechiae, twenty-two out of 31 (70.97%) facial sides reported transient petechiae on the P-MLA side, while no patient reported petechiae on the AF-Er side (*P* < 0.05).

**TABLE 3:** Adverse effects.

Adverse event	P-MLA side	AF-Er side	*P*-value
**Immediate AEs**			
Pain (VAS scale)	2.14 ± 1.00	3.53 ± 1.30	*P* < 0.05*
Erythema (0∼3 scale)	1.12 ± 0.70	0.94 ± 0.60	*P* < 0.05*
Edema, *n*	2/31	4/31	*P* < 0.05*
Exudation, *n*	2/31	30/31	*P* < 0.05*
Petechiae, *n*	22/31	0/31	*P* < 0.05*
Pinpoint bleeding, *n*	9/31	12/31	*P* = 0.30
**Long-term AEs**			
Crust shedding time, day	0.5 ± 0.68	2.21 ± 0.68	*P* < 0.05*
Duration of erythema, day	4.10 ± 1.82	2.87 ± 1.63	*P* < 0.05*
Duration of edema, day	1.32 ± 1.23	2.04 ± 1.45	*P* < 0.05*
Hyperpigmentation, *n*	1/31	8/31	*P* < 0.05*
Secondary scarring, *n*	0/31	0/31	NA
Pruritus, *n*	22/31	15/31	*P* = 0.06
Appear, day	1.49 ± 0.58	2.52 ± 0.84	*P* < 0.05*
Disappear, day	3.60 ± 1.04	4.32 ± 0.65	*P* < 0.05*
Acneiform eruption, *n*	5/31	1/31	*P* = 0.10

P-MLA, picosecond lasers with MLA handpiece; AF-Er, ablative fractional 2940-nm Er:YAG laser; AE, adverse effect; VAS, visual analog scale; NA, not available. *P < 0.05 between two treatments.

For the long-term adverse effects, both the average shedding time of crust and duration of edema were significantly shorter on the P-MLA side than on the AF-Er side (crust shedding time: 0.5 ± 0.68 vs. 2.21 ± 0.68; duration of edema: 1.32 ± 1.23 vs. 2.04 ± 1.45; both *P* < 0.05). In contrast, the duration of erythema was significantly longer in patients treated with P-MLA (4.10 ± 1.82 days) than those treated with AF-Er (2.87 ± 1.63 days; *P* < 0.05). As for PIH, only one patient complained of mild PIH on the P-MLA side, while eight experienced PIH on the AF-Er side (*P* < 0.05). Pruritus was occurred in 22 out of 31 patients on the P-MLA side with the mean onset and disappear time of 1.49 ± 0.58 days and 3.60 ± 1.04 days, respectively. On the AF-Er side, 15 out of 31 patients experienced pruritus, and the mean appearing and disappearing time was 2.52 ± 0.84 days and 4.32 ± 0.65 days, respectively. Acneiform eruption was reported in five patients on the P-MLA side (Supplementary Figure 1) and one patient on the AF-Er side, respectively. No patient experienced secondary scaring on both sides.

## 4. Discussion and conclusion

In this randomized, split-face, controlled study, we demonstrated that both P-MLA and AF-Er could significantly improve atrophic acne scars and the effects were observed as early as two treatment session. The AF-Er produced a slightly greater clinical response compared with P-MLA based on the patient’s assessments. However, the results assessed by investigators showed no significance. Overall, the two devices did not differ largely in terms of efficacy. For safety profiles, no serious side effects were reported on both sides. The P-MLA side showed lower pain level, shortened duration of crust shed and edema, and less occurrence of PIH. However, petechiae and erythema were more obvious treated by P-MLA. To the best of our knowledge, this is the first clinical study comparing the efficacy and safety of P-MLA and AF-Er for the treatment of atrophic acne scar.

A number of interventions are available to reduce the appearance of atrophic acne scars. Among them, fractional lasers including both the non-ablative and ablative types remain the first-line treatment option (7, 22). Recently, the picosecond laser has demonstrated its effectiveness in the treatment of acne scars with particular attention to dark-skinned individuals (23–25). Fractionated picosecond laser delivers micro-spots of focal high energy and spares surrounding area with low-level heat, which is absorbed by intra-epidermal melanin within the epidermal focal zone. Beneath these localized zones, an electron avalanche breakdown alternatively termed laser-induced optical breakdown (LIOB) produces vacuoles in epidermis, dermis, or both with minimal collateral thermal damage of surrounding tissues (26). In the dark skin, the laser-induced vacuolization occurred near the basal membrane, while larger vacuoles occurred at the deeper locations in the light skin (14). The process of LIOB can trigger pressure fluctuation in the dermis and increases production of dermal collagen, elastic tissue and mucin, and thereby results in dermal remodeling (27).

Several previous studies had compared the efficacy and safety of picosecond lasers with non-ablative fractional lasers. Kwon et al. treated atrophic scars on 25 Korean patients with picosecond laser and a non-ablative erbium-glass fractional laser, and found picosecond laser achieved a significantly better improvement in acne appearance with less severe pain (28). Likewise, a similar conclusion was reached by Shi et al., who comparing two devices on 22 Chinese patient (20). In addition, Shi et al. also demonstrated better outcomes in pores and skin glossiness when treated by picosecond laser. How about fractional ablative lasers? Non-ablative fractional lasers should be a less painful and traumatic procedure compared with ablative fractional lasers, while the effects obtained on atrophic scars were not as impressive as those using ablative fractional lasers (6). In the study by Sirithanabadeeku et al., the picosecond laser was demonstrated as effective as ablative fractional CO_2_ laser in treating acne scars with more safety profiles. However, no study has compared the picosecond laser with ablative Er:YAG laser in treating acne scars.

Compared to the CO_2_ laser, the Er:YAG laser is so selective for water that its action is superficial and less underlying thermal damage (29). In this study, we indicated that both devices were effective in treating atrophic acne scars. However, the mean satisfaction score was significantly higher on the AF-Er side than the P-MLA side and the patients were more satisfied with AF-Er. Therefore, AF-Er produced a slightly greater clinical response compared to P-MLA based on the patient’s assessments. Overall, the two devices did not differ largely in terms of efficacy. As for pores and skin texture, the non-invasive VISIA system was used to evaluate these skin changes, which reflecting lasers’ effect more objectively. Both P-MLA and AF-Er achieved an improvement in pore appearance (6.74 vs. 10.25%), although the outcomes of two devices obtained at the final visit were out of significance compared with the baseline according to both the VISIA system and patient’s assessment. As for skin texture, the results of VISIA system indicated that both devices could not alter skin texture. However, 45.16 and 51.61% patients reported satisfied or very satisfied of skin texture improvement when treated with P-MLA and AF-Er, respectively. In this prospective study, treatments were performed on all type atrophic scars. Both P-MLA and AF-Er were helpful in reducing V-type, U-type, and M-type atrophic scars. There were no significant differences between these two devices. The similar mechanisms of collagen remodeling and re-epithelialization involving these two devices may explain these findings.

In addition to clinical response, the risk of side effects is another factor needs to be considered in practice. For the evaluation of safety, we concluded that P-MLA provided more safety profiles compared with AF-Er in Fitzpatrick skin type II to V. Our study confirmed these conclusions as downtime and pain were significantly shortened and lighter with P-MLA. In addition, for PIH, which is one of the most common and frustrating adverse effects for many patients, the risk was also lower in the P-MLA side. A previous study suggested the skin pigment concentration was associated with the threshold for vacuole formation (14). Therefore, darker-skinned patients with higher melanin indices may easily experience LIOB with a relatively lower fluence threshold and less collateral damages, suggesting the P-MLA maybe a better option for Asian than AF-Er. However, erythema was more lasting on the P-MLA side. Moreover, 70.97% patients of the P-MLA side developed petechiae, while no patient experienced petechiae on the AF-Er side. In our opinion, extravasation of erythrocytes induced by photoacoustic wave may be the explanation for these clinical findings (26). The leaked plasma resulted from LIOB many also contain platelets and several growth factor molecules, such as platelet-derived growth factor, platelet-activating factor (PAF), and basic fibroblast growth factor (29). Among the adverse effects, another distinctive feature of P-MLA was a high frequency of pruritus, which was present in 70.97% patients. In addition, the symptom of pruritus appeared significantly earlier on P-MLA side than on AF-Er side with an average gap of 1.03 days approximately. On the other hand, pruritus was only presented in 48.39% of AF-Er side. The therapeutic mechanisms involving these two devices would help elucidate these clinical results. For P-MLA, pruritogens, such as PAF in the leaked plasma, may lead to the development of characteristic pruritus. As for AF-Er, pruritus may develop in response to growth factors released from the re-epithelization and dermal regeneration stage. Accordingly, pruritus and petechiae were characteristically observed following the picosecond laser treatment in our study, which was consistent with previous study (30).

There were some limitations in this study. First, all enrolled subjects were similar in ethnic background. Second, the follow-up duration was only 20 weeks and it should be lengthened to obtain longer-term results. Third, because of the lack of previous data, we cannot calculate sample size using a calculation method. Therefore, we performed an exploratory study with a small sample. The sample size was determined based on previous feasibility studies rather than a power analysis. Finally, the sample size was relatively small and a larger sample size may be required in the future studies.

In conclusion, both P-MLA and AF-Er were effective in treating atrophic acne scars. The AF-Er produced a slightly greater clinical response compared with P-MLA according to the patient’s assessments, although the differences from investigator’s assessment were not significant. Overall, the two devices did not differ largely in terms of efficacy. However, more safety profiles, such as a shortener downtime, less pain and fewer PIH, were obtained by P-MLA. Thus, the 1064-nm picosecond laser with MLA should be a promising therapeutic alternative for the treatment of atrophic acne scars in Asian patients, especially for those patients with darker skin who searching for minimal risks.

## Data availability statement

The raw data supporting the conclusions of this article will be made available by the authors, without undue reservation.

## Ethics statement

The studies involving humans were approved by the Human Ethics Committee of Zhejiang University School of Medicine Second Affiliated Hospital (2022-0419). The studies were conducted in accordance with the local legislation and institutional requirements. The participants provided their written informed consent to participate in this study. Written informed consent was obtained from the individual(s) for the publication of any potentially identifiable images or data included in this article.

## Author contributions

RD and SC contributed to the conception and design of study, analysis and interpretation of data. YC and YS involved in acquisition of data and implementing of the research. RD wrote the manuscript. SC gave final approval of the version to be published. All authors read and approved the final manuscript.

## Supplementary material

Supplementary Figure 1 Clinical photograph revealed acneiform eruptions from P-MLA in a 21-year-old man with Fitzpatrick skin Type V. P-MLA, picosecond lasers with MLA handpiece.
