# Supplementary figures and images for: Comparison of 1064-nm Nd:YAG picosecond laser using fractional micro-lens array vs. ablative fractional 2940-nm Er:YAG laser for the treatment of atrophic acne scar in Asians: a 20-week prospective, randomized, split-face, controlled pilot study
Source: Front Med (Lausanne). 2023 Nov 16;10:1248831. doi: 10.3389/fmed.2023.1248831 (PMC10687437; doi:10.3389/fmed.2023.1248831)

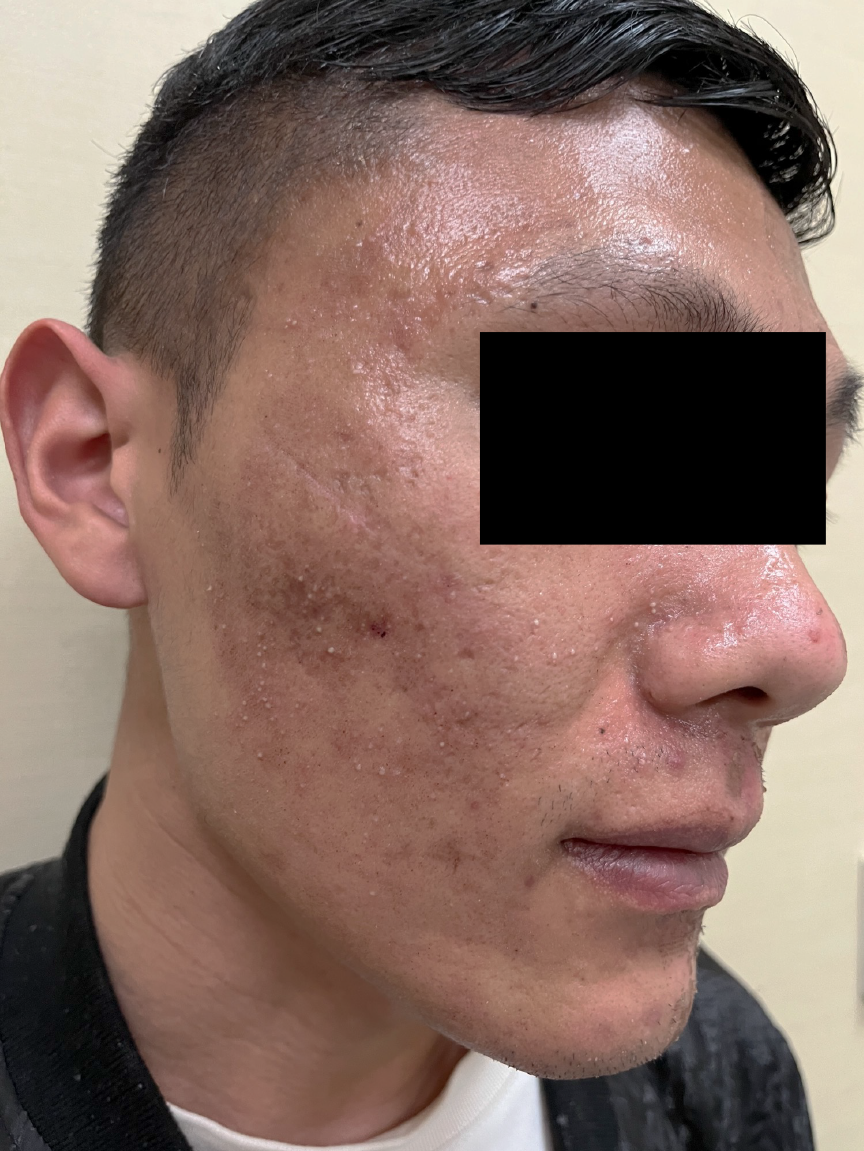

Supplement: Supplementary Figure 1 — Clinical photograph revealed acneiform eruptions from P-MLA in a 21-year-old man with Fitzpatrick skin Type V. P-MLA, picosecond lasers with MLA handpiece. [file Image_1.TIF]
